# Nitric oxide induces the distinct invisibility phenotype of *Mycobacterium tuberculosis*

**DOI:** 10.1038/s42003-024-06912-0

**Published:** 2024-09-28

**Authors:** Brindha Gap-Gaupool, Sarah M. Glenn, Emily Milburn, Obolbek Turapov, Marialuisa Crosatti, Jennifer Hincks, Bradley Stewart, Joanna Bacon, Sharon L. Kendall, Martin I. Voskuil, Olga Riabova, Natalia Monakhova, Jeffrey Green, Simon J. Waddell, Vadim A. Makarov, Galina V. Mukamolova

**Affiliations:** 1 Leicester Tuberculosis Research Group, Department of Respiratory Sciences, University of Leicester, Leicester, LE1 9HN UK; 2 FACS Facility Core Biotechnology Services, University of Leicester, Leicester, LE1 9HN UK; 3 Discovery Group, Vaccine Development and Evaluation Centre, UK Health Security Agency, Porton Down, SP4 0JG UK; 4 Centre for Endemic, Emerging and Exotic Disease, the Royal Veterinary College, Hatfield, Hertfordshire AL9 7TA UK; 5 Department of Immunology and Microbiology, University of Colorado Anschutz Medical Campus, Aurora, CO USA; 6 Research Center of Biotechnology, Russian Academy of Sciences, Moscow, Russia; 7 School of Biosciences, University of Sheffield, Sheffield, S10 2TN UK; 8 Global Health and Infection, Brighton and Sussex Medical School, University of Sussex, Brighton, BN1 9PX UK; 9 The National Institute for Health and Care Research Leicester Biomedical Research Centre, University of Leicester, Leicester, LE1 9HN UK

**Keywords:** Pathogens, Bacteriology

## Abstract

During infection *Mycobacterium tuberculosis* (Mtb) forms physiologically distinct subpopulations that are recalcitrant to treatment and undetectable using standard diagnostics. These difficult to culture or differentially culturable (DC) Mtb are revealed in liquid media, their revival is often stimulated by resuscitation-promoting factors (Rpf) and prevented by Rpf inhibitors. Here, we investigated the role of nitric oxide (NO) in promoting the DC phenotype. Rpf-dependent DC Mtb were detected following infection of interferon-γ-induced macrophages capable of producing NO, but not when inducible NO synthase was inactivated. After exposure of Mtb to a new donor for sustained NO release (named NOD), the majority of viable cells were Rpf-dependent and undetectable on solid media. Gene expression analyses revealed a broad transcriptional response to NOD, including down-regulation of all five *rpf* genes. The DC phenotype was partially reverted by over-expression of Rpfs which promoted peptidoglycan remodelling. Thus, NO plays a central role in the generation of Rpf-dependent Mtb, with implications for improving tuberculosis diagnostics and treatments.

## Abstract (web summary)

Mechanistic study reveals nitric oxide as the trigger of distinct persister-like phenotypes of *Mycobacterium tuberculosis* during infection and highlights the importance of resuscitation promoting factor for this phenomenon.

## Introduction

*Mycobacterium tuberculosis* (Mtb) is the causative agent of tuberculosis (TB), which resulted in the death of 1.6 million people in 2021^1^. Standard TB treatment lasts at least 6 months, and significant resources are directed toward developing shorter and more effective treatments^2^. One barrier to achieving this goal is the presence of Mtb populations that are often missed by standard diagnostic tests. These difficult-to-culture bacilli do not produce colonies on agar and only grow in liquid media, often requiring supplementation with culture supernatant (CSN)^3^ and are therefore known as differentially culturable (DC) Mtb^4^. DC Mtb have also been referred to as non-culturable bacteria^5^ and differentially detectable bacteria^6^. DC Mtb have a higher tolerance to some anti-TB drugs^3,7^, and drug treatment increases the proportion of DC Mtb in patients^3,6,8–10^. DC Mtb are also detectable in infected murine lungs and spleens^11,12^ and show higher resistance to rifampicin^11^. Thus, DC Mtb are believed to exist in a poorly characterized, non-replicating state, emergence from which requires liquid media or CSN for resuscitation and restoration of the ability to form colonies on solid media.

CSN contains resuscitation-promoting factor (Rpf) proteins, which are peptidoglycan-remodeling enzymes^13^ that can revive DC Mtb from the sputa of TB patients^3,14^ and from animal lungs^15^. Rpf inhibitors^16^ impair regrowth of DC mycobacteria from sputum^17^ and animal tissue^15^. Furthermore, *ΔrpfB* and *ΔrpfAB* Mtb mutants exhibited reactivation defects in C57BL/6 mice treated with the inducible nitric oxide synthase (iNOS) inhibitor, aminoguanidine^18,19^ or in iNOS^−/−^ mutant mice^19^ suggesting an interplay between iNOS and Rpfs in controlling Mtb persistence in vivo. Whilst resuscitation of these Rpf-dependent DC Mtb has been linked to TB relapse in mice^11^, the importance of this process in human TB remains to be established.

The molecular mechanism(s) underpinning the generation of DC Mtb is (are) currently unknown, although the in vivo environment has been proposed as a key factor^15^. Nitric oxide (NO) is a well-characterized component of the innate immune system that is deployed to control intracellular pathogens, like Mtb^20^. The iNOS of macrophages is critical for controlling Mtb in mice^21^ and humans^22^. NO limits Mtb intracellular growth^23^ due to its direct antibacterial effects and/or by influencing the host cell-mediated inflammatory response^22^. Compounds that release NO (NO donors) produce a range of outcomes from bacteriostatic to bactericidal when added to Mtb cultures, dependent in part on the kinetics of NO liberation from the donor^22^. Overall, results of NO exposure in vitro revealed relatively minor impacts on Mtb viability and cellular metabolism^22^ and have thus far failed to explain the significant effects of NO observed in vivo^21,23^.

The aim of this study was to evaluate the role of NO in the induction of Rpf-dependent DC Mtb and to understand molecular mechanisms underpinning Rpf dependency in mycobacteria, using macrophage infection models and a new donor for sustained exposure to NO (NOD).

## Results

### Rpf-dependent DC Mtb are produced in IFN-γ-activated murine macrophages

**Fig. 1:**
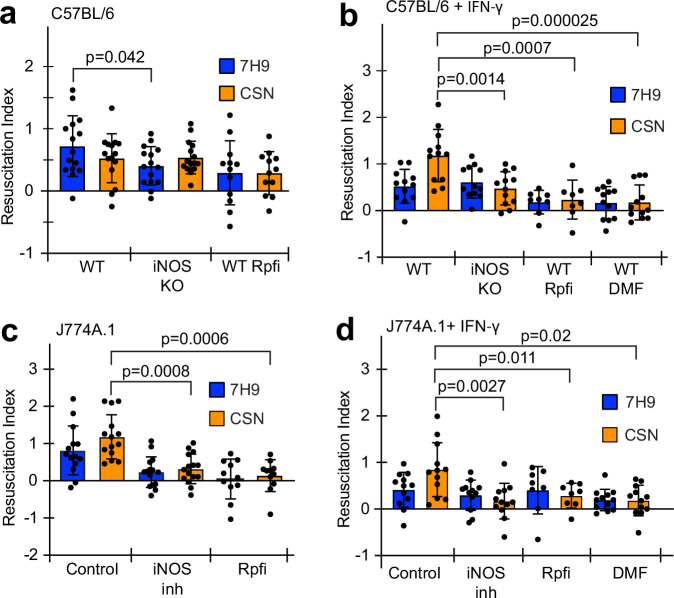
Rpf-dependent DC Mtb were formed in IFN-γ-stimulated murine macrophages with functional iNOS activity. Macrophages were infected with Mtb at MOI 1 for 24 h prior to CFU and MPN counting in 7H9 or CSN. The Rpf inhibitor (Rpfi) was added to resuscitation media as indicated. Data presented as RI values, RI = Log_10_MPN/ml−Log_10_CFU/ml. See Supplementary Fig. 1 for Mtb cell counts. **a**, **b** C57BL/6 wild type (WT) and iNOS knockout (iNOS KO); **a** untreated C57BL/6 or **b** IFN-γ treated C57BL/6. **c**, **d** J774A.1 cells; **c** untreated control or **d** IFN-γ-treated cells. iNOS was inhibited by the addition of aminoguanidine (iNOS inh). Data are means ± SEM from at least 12 biological replicates from four experiments; *p*-values are for unpaired *t*-test. Chemical concentrations used: IFN-γ, 10 ng/ml; aminoguanidine; 500 μM; DMF, 25 μM; Rpfi, 35 μM, (the concentrations of aminoguanidine and DMF were nontoxic for macrophages in accordance with previously published studies)^23,68^.

Mtb can survive and replicate in macrophages where they may be exposed to NO^24^. Murine macrophage cell lines produce NO, especially after stimulation with IFN-γ^23^. Two different murine macrophage cell lines (C57BL/6 and J774A.1) were used to investigate whether macrophage-generated NO resulted in the formation of DC Mtb intracellularly (Fig. 1 and Supplementary Fig. 1). Resuscitation Indices (RIs) reflecting the difference between Mtb regrown in liquid media (MPN measurements) and Mtb producing colonies on solid agar plates (CFU measurements) were calculated using the following formula RI = Log_10_(MPN/ml)–Log_10_(CFU/ml).

Figure 1a shows that C57BL/6 macrophages infected with Mtb for 24 h had a DC Mtb population recoverable in 7H9 medium alone, as indicated by a resuscitation index (RI) of 0.7. This population was significantly lower in the infected C57BL/6 iNOS knockout macrophages (*p* < 0.05). However, for both WT C57BL/6 macrophages and iNOS knockout C57BL/6 macrophages the RI values were not significantly greater when medium supplemented with CSN was used compared to 7H9 medium alone (Fig.1a). Moreover, application of an Rpf inhibitor (3-nitro-4-thiocyanato-phenyl)-phenyl-methanone)^16^, Rpfi, did not result in a statistically significant (*p* > 0.05) decrease in RI values. These data suggested that the DC Mtb generated under these conditions were not dependent on exogenous Rpf proteins.

In the experiments described above, nitrite (a stable product of NO oxidation^25^) was undetectable in the culture medium, indicating that NO production by C57BL/6 cells under these conditions was, at best, low. Therefore, the C57BL/6 macrophages were stimulated with IFN-γ resulting in detectable nitrite in the culture medium (2.02 ± 0.18 µM, *n* = 6 from two experiments). Now the DC Mtb population recovered in CSN (in the presence of exogenous Rpf) increased (RI = 1.2) compared to both recovery in 7H9 medium (RI = 0.52), Fig. 1b, and to Mtb recovered from unstimulated C57BL/6 macrophages (Fig. 1a). Addition of Rpfi dramatically decreased recovery of this population (RI = 0.24). Furthermore, the DC Mtb population recovered in the presence of CSN from the IFN-γ stimulated iNOS knockout macrophages was significantly smaller (*p* < 0.01) than that from stimulated WT macrophages (Fig. 1b), and resuscitation in both 7H9 and CSN supplemented media was prevented by addition of a Rpfi (Fig. 1b). These data show that Rpf-dependent DC Mtb was formed in IFN-γ-stimulated, NO-producing WT C57BL/6 macrophages. Previous studies established that DC Mtb was eliminated from infected murine organs by treatment with the anti-inflammatory compound dimethyl fumarate (DMF)^12^. This was also the case for infected IFN-γ-stimulated WT C57BL/6 macrophages, where the Mtb RI was reduced to 0.2 (Fig. 1b).

Next, the NO-dependent generation of DC Mtb in macrophages was investigated using a second cell line, J774A.1. After 24 h of infection, DC Mtb were recovered from unstimulated macrophages, but not from J774A.1 cells with chemically inactivated iNOS, using the murine iNOS inhibitor aminoguanidine (Fig. 1c). DC Mtb could be recovered in 7H9 medium (RI = 0.8), and addition of CSN improved resuscitation (RI = 1.2), which was abolished by the addition of the Rpf inhibitor (RI < 0.2). In IFN-γ-stimulated macrophages the DC Mtb population was only recoverable in CSN-containing medium (RI = 0.85), and significant DC Mtb populations were not produced after treatment with aminoguanidine (RI = 0.17), or the Rpf inhibitor (RI = 0.29) or DMF (RI = 0.27) (Fig. 1d). Collectively, our data suggest that generation of Rpf-dependent DC Mtb in macrophages can be triggered by NO and is prevented by iNOS inhibitors or the anti-inflammatory drug, DMF.

### Treatment with a novel nitric oxide donor induces Rpf dependency in Mtb and BCG

We subsequently investigated whether DC mycobacteria could be generated by exposure to NO in vitro. Mtb was incubated in 7H9 medium with acidified nitrite (10 mM) for 48 h; however, Mtb CFU counts did not substantially change (Supplementary Fig. 2a). Commercially available NO donors, Diethylamine NONOate (DETA/NO, 100 µM), and spermine NONOate (200 µM) also had no impact on Mtb CFU counts (Supplementary Fig. 2a). We next measured levels of NO released by DETA/NO and acidified nitrite. Accumulation of NO in the culture medium was assessed by measurement of nitrite, a stable oxidation product of NO^25^; intracellular NO was detected by staining with DAF-FM diacetate^26^. The addition of DETA/NO dramatically increased the concentration of nitrite in the spent medium after 1 h, but no further increase of nitrite was detected after 4 or 24 h of treatment (Supplementary Fig. 2b). A marginal increase in nitrite concentration was detected in lysates of DETA/NO-treated mycobacteria after 1 h but not after 4 h of exposure. After 24 h of DETA/NO treatment, high concentrations of nitrite were detected in mycobacterial lysates; however, the results were highly variable (Supplementary Fig. 2c). DAF-FM diacetate staining showed no significant increase of fluorescence in DETA/NO-treated mycobacteria compared with untreated control (Supplementary Fig. 2d). Thus, in our experimental system, DETA/NO appeared to release NO spontaneously and rapidly, but only a small proportion of NO penetrated the mycobacteria, and did not induce DC Mtb. Similar results were obtained with acidified nitrite (Supplementary Fig. 2).

**Fig. 2:**
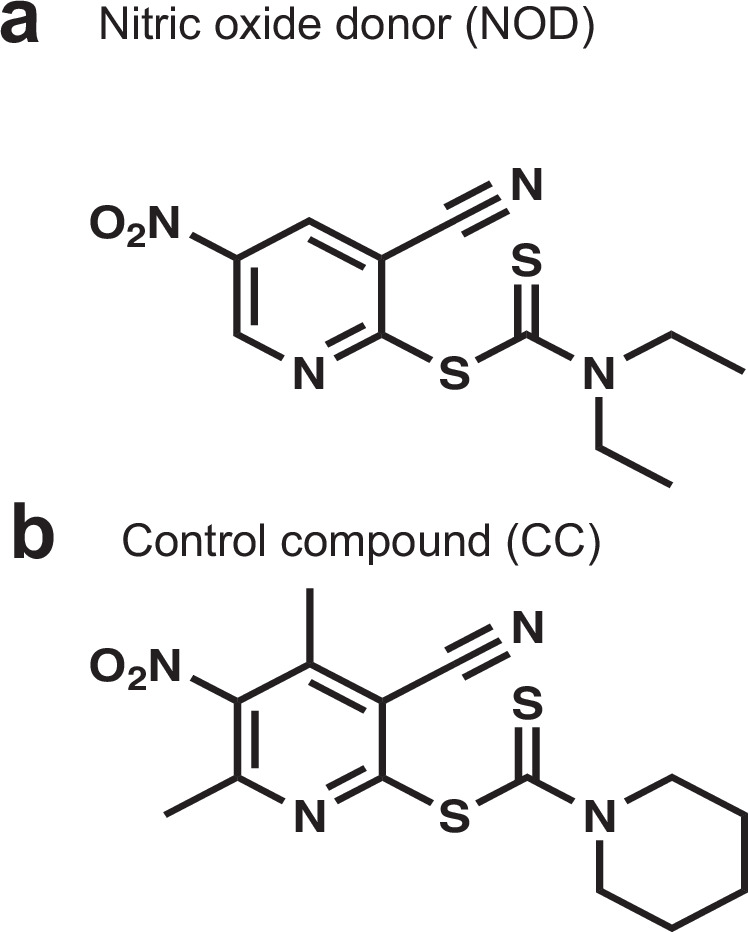
Chemical structures of the nitric oxide donor (NOD) and control compound (CC). **a** NOD 3-cyano-5-nitropyridin-2-yl diethyldithiocarbamate and **b** CC 6-dimethyl-5-nitropyridin-2-yl piperidine-1-carbodithioate.

**Fig. 3:**
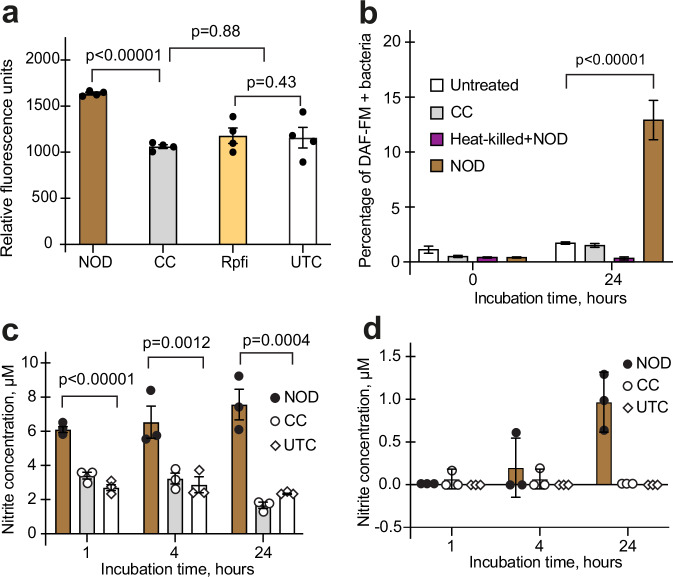
NOD enabled intracellular NO release. **a** Detection of NO in BCG using DAF-FM diacetate and fluorescence measurement. Bacteria were either untreated (UTC) or incubated with NOD, CC, or Rpf inhibitor (Rpfi) for 1 h. Data are means ± SEM for four biological replicates from one representative experiment; *p*-values are for the unpaired *t*-test. **b** Detection of DAF-FM diacetate positive cells in BCG cultures treated with either 100 µM NOD or CC for 24 h using flow cytometry. As well as UTC, the control experiments included heat-treated BCG mixed with 100 µM NOD (Heat-killed + NOD); the *p*-value is for the unpaired *t*-test. **c**, **d** Assessment of nitrite concentration in lysates (**c**) or spent media (**d**) prepared from BCG treated with either NOD or CC for 24 h, *p*-values are for one-way ANOVA. Chemical concentrations used: NOD or CC, 100 µM in **a**, **b** and 200 µM in **c**, **d**; Rpfi, 35 µM; DAF-FM diacetate, 10 µM. **b–d** Data are means ± SEM for three biological replicates from one representative experiment.

Based on these findings, we concluded that the tested NO donors were not suitable for sustained exposure to NO, which might be required for the generation of DC mycobacteria. A new NO donor (NOD), 3-cyano-5-nitropyridin-2-yl diethyldithiocarbamate (Fig. 2a) for sustained intracellular NO exposure and a structurally related control compound (CC), 3-cyano-4,6-dimethyl-5-nitropyridin-2-yl piperidine-1-carbodithioate (Fig. 2b) were designed and synthesized. NOD did not release NO spontaneously in 7H9 medium, where pH was 6.8 (Supplementary Fig. 3a); however, a rapid increase of fluorescence was observed (within 1 h) in NOD-treated *Mycobacterium bovis* BCG (BCG), but not in CC-treated mycobacteria (Fig. 3a, Supplementary Fig. 3b). Moreover, a significant DAF-FM diacetate positive BCG population could be detected after 24 h of exposure to NOD; this was not the case for CC-treated, untreated, or heat-killed BCG treated with NOD (Fig. 3b). To confirm our findings, we measured nitrite concentrations in bacterial lysates obtained from treated and untreated BCG cultures (Fig. 3c). NOD treatment resulted in significant increase of nitrite within 1 h as compared with CC-treated and untreated BCG cultures (*p* < 0.00001, one way ANOVA). Furthermore, more than 50% of cells in NOD- and CC-treated samples were propidium iodide negative and SYTO 9 positive, and, therefore, likely remained viable (Supplementary Fig. 3c). Accumulation of small amounts of nitrite was detected in the spent medium of NOD-treated BCG only after 24 h (Fig. 3d). These observations suggested that NOD entered live BCG, releasing measurable levels of NO without causing bacterial cell lysis. Therefore, NOD was deemed suitable for testing the effects of NO exposure on growth phenotypes using CC as a control.

**Fig. 4:**
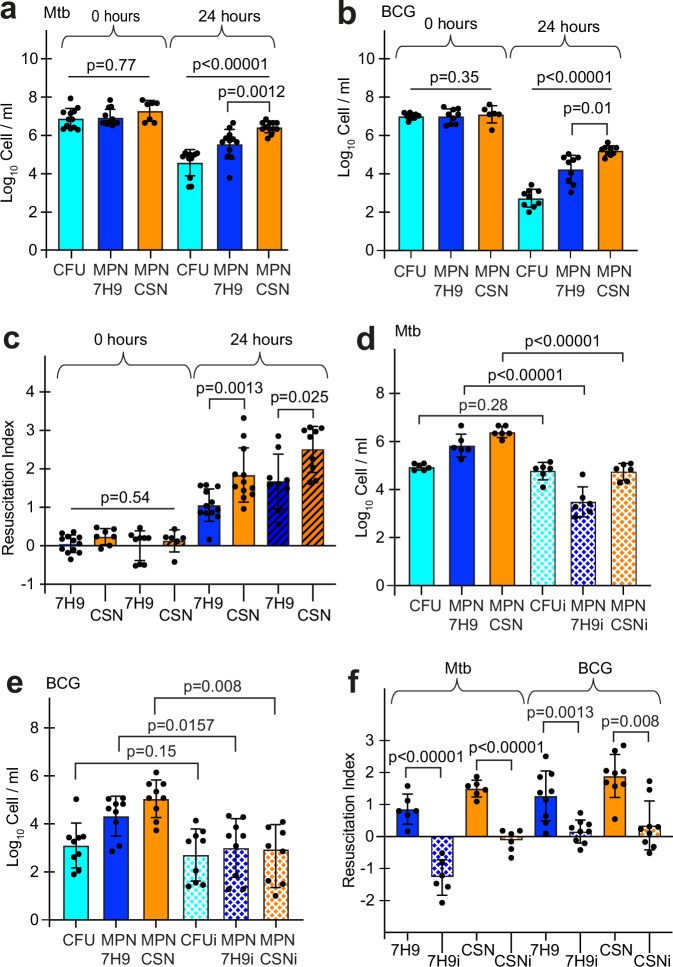
NOD treatment induced Rpf-dependent mycobacteria in vitro. **a–f** Mtb or BCG were treated with 100 µM NO donor (ND) for 24 h at 37 °C without shaking. CFU, MPN 7H9, and MPN_CSN counts for Mtb (**a**, **d**) and BCG (**b**, **e**) were determined. **c** Resuscitation indices (RI) were calculated for Mtb (filled bars) and BCG (hatched bars). **f** RI was calculated for Mtb and BCG samples resuscitated with Rpfi (dotted bars) or without Rpfi. **d–f** Rpfi (35 µM) was added to resuscitation media, 7H9 or CSN (“i”—resuscitation in the presence of Rpfi), and cell counts were determined for Mtb (**d**) or BCG (**e**). **a**, **b**, **d**, **e** *p*-values are for one-way ANOVA; **c,** **f** *p*-values are for unpaired *t*-test. Data are means ± SEM for at least six replicates from at least two experiments. RI = Log_10_MPN/ml−Log_10_CFU/ml.

While incubation of Mtb with 100 µM CC for 24 h had no effect on cell counts in liquid or on solid media (Supplementary Fig. 4a), exposure to NOD resulted in a 100-fold reduction in CFU counts (Fig. 4a). In liquid 7H9 medium the recovered cell counts were ~10-fold higher, and supplementation with CSN further increased this number >10-fold (Fig. 4a). More detailed analysis revealed a gradual decline of CFU counts in cultures of NOD-treated Mtb over 48 h (Supplementary Fig. 4b). This suggested that, like Mtb from clinical TB samples, most of the NOD-treated cells were DC Mtb^3,4^. Similar results were obtained with BCG (Fig. 4b). BCG cultures had lower CFU counts after 24 h compared to Mtb (Fig. 4a), and resuscitation indices (difference between CFU and MPN counts) were slightly higher in BCG compared with Mtb cultures (Fig. 4c). As might be expected, differences were observed between experiments carried out at different times with independent cultures; however, the RI values for NOD-treated mycobacteria measured in the presence of CSN were always greater than those obtained with unsupplemented 7H9 medium (Fig. 4c). Thus, these results revealed that NOD treatment of both Mtb and BCG led to the formation of DC bacteria.

The addition of the Rpfi to resuscitation media (7H9 or CSN), abolished the resuscitation of NOD-treated Mtb (Fig. 4d) or BCG (Fig. 4e). Figure 4f shows RI values calculated for NOD-treated Mtb and BCG resuscitated in media with or without Rpfi and confirms that Rpfi greatly reduced resuscitation of both mycobacteria. In accordance with previous findings^16^, the Rpfi had no effect on actively growing Mtb (Supplementary Fig. 5). Rpfi also did not increase DAF-FM diacetate fluorescence (Fig. 3a), suggesting that it did not release NO.

### NOD treatment produced a broad transcriptional response and down-regulated expression of *rpf* genes

**Fig. 5:**
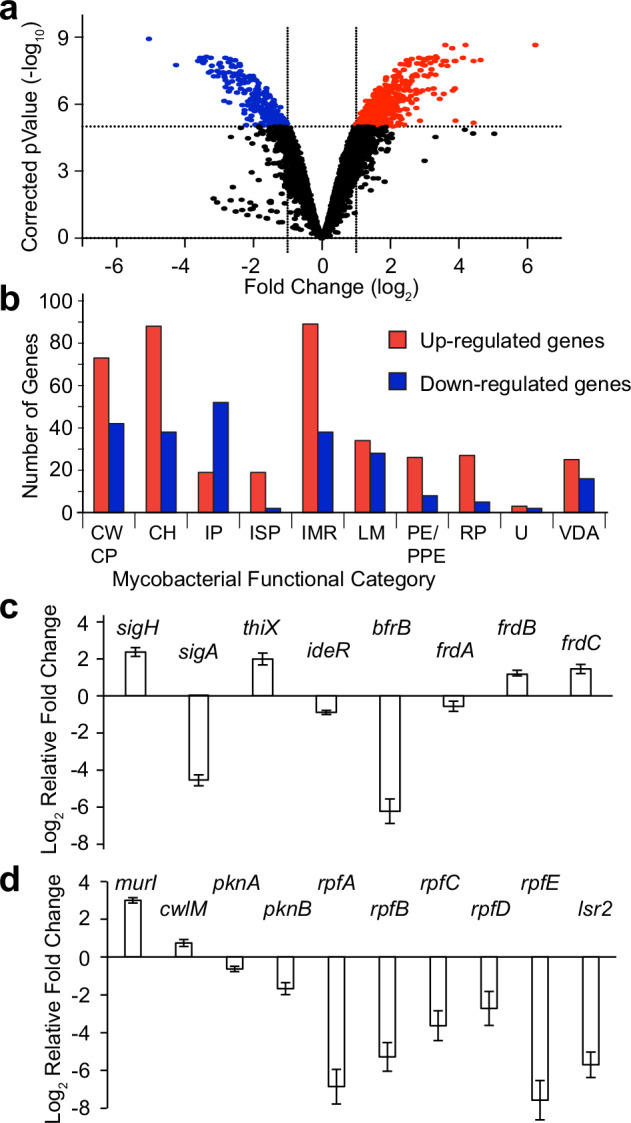
Mtb exposure to NOD for 4 h led to activation of several regulatory pathways. **a** Volcano plot showing 407 genes significantly induced (red) and 233 genes repressed (blue) by NOD compared to CC at 4 h. Significantly differentially expressed genes (DEG) were identified using a moderated *t*-test (*p* < 5 × 10^−5^ with Benjamini and Hochberg multiple testing correction) and fold change >2.0. **b** Distribution of DEG from functional categories^69^. CWCP cell wall and cell processes, CH conserved hypothetical, IP information pathways, ISP insertion sequences and phages, IMR intermediary metabolism and respiration, LM lipid metabolism, RP regulatory proteins, U unknown, VDA virulence, detoxification, adaptation. **c**, **d** Expression of selected genes was confirmed by RT-qPCR, normalized to 16S rRNA transcript abundance. Log_2_ relative fold difference was calculated. Data are means ± SEM for three independent cultures done in technical duplicates (*n* = 6).

To investigate molecular mechanisms controlling the formation of DC Mtb in response to treatment with NOD, transcript-profiling experiments were conducted. Cultures were sampled 4 h after exposure to either NOD or CC. At this time, for NOD-treated cultures, the CFU count was decreasing, but the MPN_CSN value was not, suggesting that the transition to the DC state had begun (Supplementary Fig. 4b). Compared to treatment with CC, 640 (407 induced, 233 repressed) genes were differentially expressed, fold change >2, *p* < 5 × 10^−5^ (Fig. 5a, Dataset 1). The changes in gene expression spanned a wide range of functional categories (Fig. 5b). The DosR regulon that has been previously shown to react to hypoxia and NO, signals associated with non-replicating states of Mtb^27–30^, was not induced after NOD treatment (Dataset 1). Moreover, the Mtb *dosR* deletion mutant, a complemented strain, and the parent Mtb treated with NOD for 24 h resulted in similar numbers of DC Mtb; all strains were equally resuscitated in CSN-supplemented medium (Supplementary Fig. 6).

Several transcriptional changes indicated adaptation to impaired aerobic respiration (Dataset 1). These included the up-regulation of fumarate reductase (*frd*) genes (Fig. 5c), suggesting the utilization of fumarate as a terminal electron acceptor to maintain redox balance and membrane potential^31^, and the up-regulation of the *pta-ackA* operon, coding for phosphotransacetylase and acetate kinase, indicating increased reliance on substrate-level phosphorylation for ATP generation (Dataset 1). We note that expression of *icl1*, coding for isocitrate lyase, an enzyme of the glyoxylate cycle that is critical for survival under hypoxia^32^, was down-regulated (6.15-fold) in response to NOD treatment (Dataset 1). Like the absence of a DosR regulon response, the down-regulation of *icl1* contrasts with several other studies of NO-treated Mtb in which *icl1* was up-regulated^29,30^. Taken together, these differences in gene expression suggest that NOD induces distinct physiological adaptations compared to other models. However, we note that previous studies showing strong induction of the DosR regulon by NO used well-aerated cultures, conditions that likely result in initial suppression of the DosR regulon. In contrast, here, to better mimic the in vivo environment, we used static Mtb cultures in which the DosR regulon is likely to be already, at least partially, induced^28^. Our data support proteomic data reported previously suggesting that the abundance of DosR regulon proteins declined during prolonged NO treatment after relatively modest initial up-regulation^30^.

The 407 up-regulated genes were analyzed using the transcription factor overexpression (TFOE) tool^33^ to identify transcription regulators that mediate the response to NOD treatment. Seven transcription regulators passed the significance threshold (TFOE output: significance of enrichment of their regulons *p* < 5 × 10^−5^), including three alternative sigma factors (SigH, SigF, and SigK) and four transcription regulators (Rv0047, WhiB2, Rv2175, and FurA, Dataset 2). SigH and SigF were also prominent (significance *p* < 0.05) in the ChIP-seq output (Dataset 2). SigH coordinates the response to oxidative, nitrosative, and heat stresses in Mtb, and *sigH* expression is induced in macrophages, reviewed by Manganelli^34^. Additionally, SigH is activated by oxidation of its anti-sigma factor RshA, which can be triggered by NO. SigF is associated with cell envelope integrity and is controlled by anti-sigma factor agonists, RsfA and RspB, which sequester the anti-sigma factor RsbW, and also by the anti-sigma factor Rv1364c. SigK is likely involved in maintaining the redox state of the mycobacterial periplasm^34^.

The *sigH* and *sigF* genes were up-regulated (3.82-fold and 5.79-fold, respectively; Dataset 1, Fig. 5c), whereas expression of *sigK* was not significantly changed. The up-regulation of *sigH* and *sigF* was accompanied by down-regulation of the primary sigma factor, *sigA* (4.56-fold), consistent with increased occupation of core RNA polymerase by alternative sigma factors and redistribution of the transcriptional machinery in response to NOD (Dataset 1, Fig. 5c).

The involvement of SigH, SigF, and SigK in the response to NOD suggests that Mtb was exposed to oxidative, nitrosative, and cell wall stresses during NOD-induced transition to Rpf-dependency. Accordingly, expression of antioxidant genes, such as the SigH/AosR-activated non-canonical cysteine biosynthetic genes (*mec-cysO-cysM*), ergothioneine biosynthesis genes (*rv3700c* and *rv3704c*), sulfate-containing compound ABC transporter (*cysA1*), sulfate adenyltransferase (*cysDN*), methionine sulfoxide reductases (*msrA* and *msrB*), rhodanese domain protein (*rv1674c*), thioredoxin reductase and thioredoxin proteins (*trxB2-C*, *thiX*, *trxB1*) were up-regulated. These antioxidant responses are consistent with adaptation to manage NO-mediated oxidative damage.

As a radical, NO reacts with transition metals such as iron resulting in damage to iron–sulfur clusters and perturbation of redox balance^35^. Disrupted iron homeostasis was suggested by the up-regulation of mycobactin biosynthetic genes (*mbtE*, *mbtG*, and *mbtH*) and ESX-3 genes required for siderophore-mediated iron uptake (*rv0282-rv0292*), and down-regulation of *ideR* repressor and iron storage systems, *bfrB* (Dataset 1, Fig. 5c).

The TFOE analysis suggested that the essential regulator of septation and cell division, WhiB2, plays a role in Mtb adaption to NO exposure (Dataset 2). Depletion of WhiB2 results in filamentous growth, whereas overexpression of *whiB2* causes hyper-septation (reviewed by Bush^36^). WhiB2 has a redox sensitive iron-sulfur cluster that is likely to react with NO, resulting in altered interaction with DNA and partner proteins^36^. Mutations that increased expression of *whiB2* shortened post-antibiotic recovery time of Mtb, as measured by the appearance of colonies on solid medium^37^, implying that the down-regulation of WhiB2 regulon genes observed here could promote the formation of DC Mtb.

In contrast to the up-regulated genes, the TFOE analysis^33^ did not identify any significantly (*p* < 5 × 10^−5^) enriched regulons that were down-regulated by NOD exposure (Dataset 2). However, the two most significant hits from the ChIP-seq output were Rv2034 (*p* = 0.017) and MtrA (*p* = 0.020).

The up-regulation of *murI*, coding for glutamate racemase, and *cwlM*, an essential regulator of peptidoglycan synthesis^38^, indicated that remodeling of the cell wall was involved in the transition to Rpf-dependency (Dataset 1, Fig. 5d). The up-regulation of *murI* is likely to be mediated by SigH (overexpression of *sigH* increased *murI* expression)^34^ and expression of *cwlM* was previously shown to be up-regulated by treatment with spermine NONOate^39^.

Consistent with the observation that NOD treatment resulted in the appearance of Rpf-dependent mycobacteria, three *rpf* genes were down-regulated (*rpfA, rpfB*, and *rpfE*; 9.5-, 3.9- and 33.2-fold, respectively) in the transcriptomic dataset (Dataset 1). This response was confirmed by RT-qPCR, which showed that all five *rpf* genes were down-regulated in NOD-treated Mtb (Fig. 5d). Expression of Rpf coding genes is tightly controlled by multiple regulators^40^, including Lsr2^41^ which was also down-regulated in NOD-treated Mtb.

### Ectopic expression of *rpf* genes impairs NOD-induced generation of DC Mtb

**Fig. 6:**
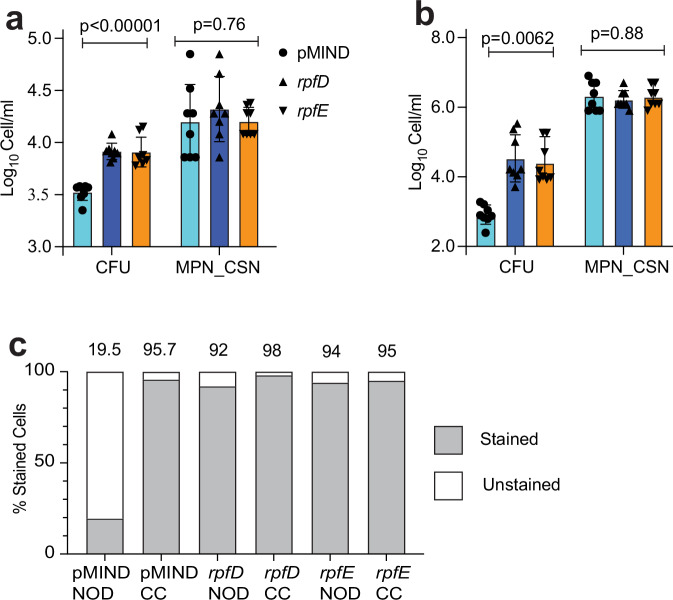
Over-expression of RpfD or RpfE partially restored Mtb culturability. **a** IFN-γ-stimulated J774A.1 cells were infected with Mtb containing either pMIND or pMIND::*rpfD* or pMIND::*rpfE* at MOI 1. CFU and MPN_CSN counts were determined at 24 h of infection. **b** Mtb containing either pMIND or pMIND::*rpfD* or pMIND::*rpfE* were treated with 100 μM NOD for 48 h at 37 °C without shaking. Data are means ± SEM for 8 in **a** and 6 in **b** biological replicates from two experiments; *p*-values are for one-way ANOVA. **c** Rpf-overexpressing strains retain the ability to incorporate HADA after treatment with NOD. For each treatment at least 100 cells were analyzed and scored by visual inspection. % stained bacteria=stained cells/total cell count*100.

Transcript profiling and RT-qPCR analyses suggested that exposure of Mtb to NOD resulted in lower expression of all five *rpf* genes. Our proteomics analysis showed that RpfA, RpfB, and RpfD were present in the Mtb CSN (Table 1). Furthermore, RpfA, RpfC, and RpfE proteins have been previously detected in Mtb CSN^42^. Thus, it was suggested that down-regulation of *rpf* genes in response to NO was important for the generation of Rpf-dependent DC mycobacteria. To test this hypothesis, we constructed Mtb strains in which expression of either *rpfD* or *rpfE* was placed under the control of a tetracycline-regulated promoter, removing them from their native regulatory networks to permit sustained expression in the presence of NOD by addition of the inducer, anhydrotetracycline. The *rpfD* and *rpfE* genes were chosen for these experiments because reliable over-expression of both had been confirmed by RT-qPCR and western blot experiments in previous studies^43^. Furthermore, RpfD has been detected in CSN, and recombinant RpfE resuscitated DC Mtb from sputum^3,14^. Macrophage infection experiments showed that strains overexpressing *rpfD* or *rpfE* had significantly higher CFU counts compared to the empty vector control (*p* < 0.001), while the numbers of resuscitating bacteria for all three strains were similar (Fig. 6a). Moreover, ectopic expression of *rpfD* or *rpfE* resulted in a ~100-fold increase in CFU counts compared with the empty vector control after NOD treatment (Fig. 6b), demonstrating partial restoration of Mtb culturability. Together, these data support an important role for NO-mediated down-regulation of *rpf* expression in the formation of DC Mtb, consistent with their Rpf-dependency phenotype. To investigate whether Rpf over-expression impacted cell wall synthesis or remodeling we used HADA, a fluorescent D-amino acid analog probe, for labeling of peptidoglycan. This approach allowed us to follow both D,D-transpeptidase-mediated peptidoglycan biosynthesis and L,D-transpeptidase-mediated peptidoglycan remodeling^44^. Treatment with NOD led to a dramatic decrease of HADA incorporation in the cell walls of Mtb carrying the control plasmid (pMIND), while most CC-treated bacteria were labeled with HADA (Fig. 6c, Supplementary Fig. 7). Importantly, HADA was still incorporated in cell walls of *rpfD* or *rpfE* overexpressing Mtb strains treated with NOD (Fig. 6c, Supplementary Fig. 7), indicating remodeling rather than biosynthesis of peptidoglycan in growth non-permissive conditions. It is suggested that this peptidoglycan remodeling is likely to promote Mtb survival and the ability to form colonies on agar without the need for exogenous application of resuscitation-promoting factors (e.g. CSN supplementation). Partial restoration of Mtb culturability could be due to sub-optimal expression of Rpfs or the necessity for all five, rather than a subset of Rpfs for rapid revival after NO stress.

**Table 1:** Rpf peptides detected in Mtb CSN

Protein	Gene	Predicted molecular weight, Da	Number of peptides detected in CSN	Sequence of peptides
RpfA	*Rv0867c*	39974.7	4	(A)ATDGEWDQVAR(C)^a^ (R)EQQIAVGER(V) (R)GAWPVCGR(G) (R)GLSNATPR(E)
RpfB	*Rv1009*	38078.4	3	(R)DDLYPAAGVQVHDA(D) (R)VEDPEMNMSR(E) (I)VEENGFSVDDR(D)
RpfD	*Rv2389c*	15678.5	1	(F)LAAETGGCSGSR(D)

^a^Residues in brackets were not detected and shown as possible cleavage site.

## Discussion

During infection pathogenic mycobacteria produce heterogeneous populations that differ in their metabolic activity, growth characteristics, and tolerance to antimicrobials (reviewed by Chung et al.)^45^. Amongst these populations, differentially culturable (DC) mycobacteria are of particular interest because they are difficult to detect and eradicate, and frequently represent the dominant Mtb population in clinical TB samples^3,4,46–48^. By genetic or chemical manipulation of NO production by two macrophage cell lines, along with the application of exogenous Rpf proteins (as CSN) and the use of a specific Rpf inhibitor, we have shown that NO induces the generation of Rpf-dependent DC Mtb in macrophages. These observations suggest that host NO (and/or its congeners) triggers this adaptive response that might be important for TB pathogenesis. Further experiments using animal models and Mtb strains with altered Rpf expression will aid in understanding Rpf's contribution to this response.

In previous work, various strategies have been developed to study the formation of DC Mtb in vitro, including prolonged incubation in stationary phase^5^, gradual acidification^49^, alterations of sodium–potassium ratio^50^, treatment with antimicrobials^11,43,51^ or inducers of oxidative stress^52^, to study the formation of DC mycobacteria in vitro. Here we describe a model system for generating DC mycobacteria (both in Mtb and BCG) based on a new NO donor—NOD (3-cyano-5-nitropyridin-2-yl diethyldithiocarbamate) that simulates the in vivo environment by generating heterogeneous mycobacterial populations, thereby permitting investigation of the physiologically distinct states produced during the pathogenesis of TB. Our data suggest that NOD treatment results in the release of NO in living bacteria; however, the biochemistry of NO is complex^53^. For example, whilst our experiments using DAF-FM diacetate strongly suggest the presence of detectable NO associated with NOD-treated mycobacteria, the presence of nitrite in NOD-treated cultures suggests that there is sufficient NO and molecular oxygen available for NO oxidation, presumably by the *bcc–aa3* complex^54^ (Fig. 3). The presence of O_2_ in our cultures could potentially support superoxide generation leading to production of peroxynitrite, which is able to react with a wide range of macromolecules^55^. Moreover, NO can react with nitrite to form the oxidant, dinitrogen trioxide, which can act as a nitrosating agent^55^. Therefore, further experiments will be needed to determine the precise mechanism of NO release from NOD in mycobacterial cells, including any cellular factors involved, and the roles of NO congeners (e.g. NO^•^, NO^+^, NO^−^, ONOO^−^) in promoting the generation of DC Mtb. Nevertheless, a low active concentration of NOD in comparison with other NO donors, direct detection of NO in NOD-treated mycobacteria even after 24 h of incubation, and the availability of a structurally similar control compound make NOD advantageous for the investigation of bacterial physiology. We tested commercially available donors, however, they did not induce DC Mtb, presumably because of the rapid spontaneous release of NO in the experimental setup used in this study.

In our NOD-induced system, transition to the DC state was likely mediated by two alternative sigma factors, SigH and SigF, and their regulons, previously implicated in adaptation to redox stress and respiration-inhibitory conditions^34^. Overall, the transcriptomic signature of NOD-treated Mtb was associated with inhibition of cell division, repair of NO-induced damage, and metabolic reprogramming to restore redox balance and membrane potential. Whilst there was overlap between the transcriptional response to NOD treatment and those reported for other NO donors, there were several distinctive features associated with NOD-mediated formation of DC Mtb, including the absence of a DosR response and the down-regulation of *icl1*. These changes were also observed in Mtb surviving prolonged multi-drug treatment in mice for 28 days^56^. Remarkably, 4 out of 5 *rpf* genes (*rpfB-E*) were also downregulated in the multi-drug treatment model, while *murI* was up-regulated, similarly to the NOD-treated Mtb. We have previously shown that DC Mtb were the dominant population in mice treated with a combination of rifampicin, isoniazid, and pyrazinamide for 28 days^12^, therefore these transcriptomic adaptations might be attributed to DC Mtb. Interestingly, the transcriptional response to NOD treatment included genes from the recently identified signature of differentially detectable (DD) Mtb from sputum samples^57^. In particular, five genes, *icl1*, *ppsA*, *hspX*, *rv1738*, and *pks15*, were significantly down-regulated in sputum Mtb DD^57^ and NOD-treated Mtb (Dataset 1). Furthermore, *icl1* was down-regulated in another in vitro model for DD Mtb (the PBS-RIF model), and increased numbers of DD bacilli were formed by an *icl1* mutant^52^. Together these observations suggest that down-regulation of *icl1* and the glyoxylate cycle might be an important regulatory and metabolic adaptation in response to multiple stimuli for the formation of DCB both in vitro and in vivo.

Although the deployment of stress response systems and changes in metabolic mode are likely to be important adaptations in the transition to the DC state, one of the most striking transcriptional changes observed was the down-regulation of all five *rpf* genes in Mtb cultures treated with NOD. Whilst there is an ongoing debate concerning the importance of NO in controlling Mtb infection in humans, even though NO is detected in TB patients^58,59^, our data suggest that NO contributes to the generation of DC Mtb by down-regulation of *rpfA-E* expression. Rpfs are cell wall-cleaving enzymes, and their activities under conditions when the peptidoglycan-producing machinery is likely to be damaged could be fatal for mycobacteria as a result of uncontrolled cell lysis. Accordingly, expression of Rpf coding genes is controlled by multiple regulators^40^ and involves post-translation regulations^60–62^; however, at this time, which of these contributes to the down-regulation of these genes upon exposure to NO is unknown. Nevertheless, based on the evidence presented here, the simplest explanation for the observed Rpf-dependency of DC Mtb recovered from macrophages or after treatment with NOD is that exposure to NO down-regulates *rpf* gene expression such that mycobacteria fail to initiate Rpf production when grown in standard media. This means that emergence from the DC state requires exogenous supplementation with recombinant Rpf or Rpf-containing CSN to restart growth and peptidoglycan biosynthesis. This failure to initiate *rpf* expression could be caused directly by the action of NO on transcriptional regulatory networks or indirectly, via metabolic reprogramming in response to NO. In addition, NO also has the potential to directly damage Rpf proteins, which require a disulfide bond for correct folding and activity. The precise mechanism of Rpf-mediated resuscitation is currently unknown. HADA incorporation data reported here suggest that Rpfs may drive peptidoglycan remodeling which prevents transition to DC Mtb, however additional studies are required to investigate this observation. We cannot exclude that the Rpf effect is indirect and that resuscitation involves products of peptidoglycan degradation or other unknown factors. For example, in addition to Rpf proteins, various other molecules, including cAMP, muropeptides, phospholipids, and peptides derived from Rv1174c have been proposed to resuscitate DC mycobacteria (summarized by Dartois et al.^63^). However, none of these additional factors have been systematically validated for resuscitation of DC mycobacteria from sputum in the way that Rpf proteins have. Indeed, CSN obtained from the quintuple *rpf* deletion mutant had variable resuscitation effects^3,4^. Moreover, there is currently no published data on the composition of CSN from the quintuple *rpf* deletion mutant to suggest the identities of any alternative resuscitation factors. These observations serve to highlight the complexity of Mtb resuscitation and the need for further research to uncover the roles of any Rpf-independent resuscitation factors in that process.

**Fig. 7:**
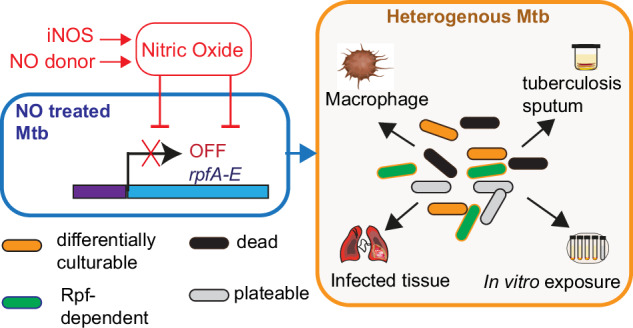
NO-mediated generation of Rpf-dependent DC Mtb. NO exposure in vivo and in vitro shuts down expression of *rpfA, rpfB, rpfC, rpfD* and *rpfE*. This leads to generation of heterogenous Mtb subpopulations including Rpf-dependent Mtb. This population heterogeneity mimics in vivo observations where NO is produced by the inducible NO synthase (iNOS) and drives formation of Rpf-dependent and differentially culturable Mtb that can be detected in infected tissue and sputum samples.

Overall, our findings suggest that differential culturability is a survival mechanism that allows mycobacteria to cope with prolonged host-imposed stresses and that this has implications for the development of better TB therapies and diagnostic tools. We hypothesise that exposure to various levels of NO drives Mtb population heterogeneity in the infected host (Fig. 7). These heterogeneous populations have different growth requirements, respond differently to drugs, and may induce differential immune responses. Here, we show that NO down-regulates the expression of all *rpf* genes and generates Rpf-dependent DC Mtb intracellularly in macrophages and in a new NO donor model for studying the transition to differential culturability. Thus, our work provides a foundation for further investigations to understand the molecular mechanisms that underpin this fundamental adaptive process and elucidate the precise role of DC Mtb in TB pathogenesis and treatment outcomes.

## Methods

### Materials and Strains

Liquid cultures were grown at 37 °C with shaking (100 rpm) for Mtb H37Rv and static for *M. bovis* BCG Glaxo in Middlebrook 7H9 broth, supplemented with 0.2% (v/v) glycerol, 10% (v/v) albumin-dextrose-catalase (ADC), and 0.05% (w/v) Tween 80 (hereafter 7H9 medium). Culture supernatants (CSN) were prepared from exponentially growing cultures (OD_580nm_ of 0.6–0.8) and sterilized by double filtration (0.22 µm filters). For MPN assays, 7H9 medium was supplemented with 50% sterile CSN. The Rpf inhibitor (3-nitro-4-thiocyanato-phenyl)-phenyl-methanone^16^) was added to resuscitation media to a final concentration of 35 µM; a concentration based on previously published data^15–17^. Mtb strains for overexpression *rpfD* and *rpfE* were obtained by electroporation of previously generated constructs^43^; expression of *rpf* genes was induced by addition of anhydrotetracycline (20 ng/ml). The *dosR* deletion mutant and complement strain were previously described^64^. Mtb stocks for macrophage infection were prepared from exponentially growing cultures (OD_580nm_ ~ 0.4–0.5). J774A.1 (ATCC® TIB-67™), C57BL/6 wild type and iNOS knockout mutant (Kerafast) cell lines were grown at 37 °C with 5% CO_2_ in Dulbecco’s Modified Eagle’s medium (DMEM) supplemented with 10% (v/v) heat inactivated fetal bovine serum. Interferon-γ (IFN-γ), dimethyl fumarate (DMF), and aminoguanidine were added to final concentrations of 1 ng/ml, 25, and 500 µM, respectively.

### Macrophage infection

Murine macrophages (C57BL/6 or J774A.1) were seeded at 2 × 10^5^ cells/ml in 24-well plates (Greiner Bio-One). IFN-γ was added 24 h prior to infection; aminoguanidine or DMF were added just before infection. The macrophages were infected with Mtb at a multiplicity of infection (MOI) of 1 and incubated at 37 °C with 5% CO_2_ for 3 h, followed by treatment with 200 µg/ml amikacin for 1 h. Amikacin was removed by washing infected monolayers with PBS twice and fresh medium containing relevant reagents was added before incubation for a further 24 h. For CFU and MPN counts, the infected macrophages were washed with PBS and lysed with 0.1% (v/v) Triton X-100.

### Assessment of viable counts

MPN counts were quantified as described before^3,7^. Mtb or BCG cells were serially diluted in 48-well microplates by adding 50 µl of cell samples to 450 µl of resuscitation medium (7H9 or CSN). For each condition, 4 replicate wells were used to calculate an MPN count for one biological sample using the MPN calculator^65^. For CSN preparation Mtb cultures were grown in roller bottles for OD_580_ 0.6–0.8, then centrifuged at 4000×*g* for 20 min. Supernatants were filter-sterilized twice using VWR 0.22 µm units. CSN aliquots (25 ml) were dried in TPP bioreactor tubes in an SP Scientific Advantage freeze drier. Dried CSN was stored at −80 °C for up to 6 months. On the day of experiments CSN was reconstituted in 25 ml sterile water and after incubation on ice for 30 min used for experiments. CSN was diluted with 7H9 (1:1). Refreezing of reconstituted CSN or additional filtering resulted in loss of resuscitation activity. MPN plates were sealed with polyvinyl tape to prevent drying, placed in double clip-lock bags, and incubated in clip-lock boxes at 37 °C without shaking for up to 12 weeks. The MPN calculator program was used for determination of MPN counts^66^.

For CFU counts, 10 µl drops from each well corresponding to 10^−1^–10^−5^ dilutions from the MPN plates (7H9 and CSN) were spotted onto Middlebrook 7H10 agar plates supplemented with 10% (v/v) ADC and 0.5% (v/v) glycerol. For each condition 4 technical CFU counts from the 7H9 MPN plate and four technical CFU counts from CSN plate were assessed. In some experiments 7H10 agar was supplemented with 0.4% (w/v) charcoal; however, it did not increase CFU counts. CFU in 7H9 and CFU in CSN were similar and for clarity, a mean value of eight technical replicate values was used for each biological replicate, unless indicated otherwise. CFU plates were placed in double clip-lock plastic bags and incubated at 37 °C for up to 12 weeks. Resuscitation Index (RI) was defined as RI = Log_10_ MPN/ml−Log_10_ CFU/ml. Limits of detection for CFU and MPN counts were 24 CFU/ml and 4.6 cells/ml, respectively. For assessment of viable counts LIVE/DEAD^TM^ *Bac*Light^TM^ Bacterial Viability kit (Thermo Fisher Scientific) and flow cytometry were used. Stained BCG samples were analyzed using FACSAria flow cytometer and BD FACSAria^TM^ software (BD Bioscience). The following parameters were applied: SYTO 9 excitation/emission at 480/500 nm; Propidium Iodide excitation/emission at 490/635 nm. Ten thousand events were recorded from each sample; single parameter fluorescence overlay plots and dual parameter dot plots were generated. Heat-killed BCG cells were included as a control. Supplementary Fig. 8 exemplifies the gating strategy. Numerical source data are available in Dataset 3 file.

### Synthesis of NO donor (NOD) and control compound (CC)

All reagents and solvents were purchased from commercial suppliers and used without further purification. ^1^H and ^13^C Spectra were measured on Bruker AC-500 (500 MHz, ^1^H) or Bruker AC-200 (75 MHz, ^13^C). Chemical shifts were measured in DMSO-d6 or CDCl3, using tetramethylsilane as an internal standard, and reported as units (ppm) values. The following abbreviations are used to indicate the multiplicity: s, singlet; d, doublet; t, triplet; m, multiplet; dd, doublet of doublets; brs, broad singlet; brm, broad multiplet. HRMS: spectra were recorded on an Agilent 1290 Infinity II HPLC system coupled to Agilent 6460 triple-quadrupole HRMS: spectrometer equipped with an electrospray ionization source. The chromatographic separation was carried out on Agilent Eclipse Plus C18 RRHD column (2.1 × 50 mm, 1.8 µm) at 40 °C with sample injection volume of 0.2 µl. The mobile phase comprising 0.1% formic acid/water (A), and 0.1% formic acid and 85% acetonitrile/water (B) was programmed to do a gradient elution (0.0–3.0 min, 60% B; 3.0–4.0 min, 60–97% B; 4.0–6.0 min, 97% B; 6.0–6.1 min, 97–60% B) at a flow rate of 0.4 ml/min. The HRMS: spectrometric detection was operated in a positive ion mode. Optimal parameters were capillary voltages of 3500 V, a nebulizer pressure of 35 psi, a gas temperature of 350 °C, a gas flow rate of 12 L/min. Purity of all compounds was measured by analytical high-performance liquid chromatography (HPLC) on an Elute HPLC system (Bruker Daltonik) equipped with Azura UVD 2.1S UV detector (Knauer) using Acquity HSS T3 column (2.1 × 100 mm, 1.3 µm, 100 Å) at 30 °C, 2 µl injection, 250 µl/min gradient elution 30─95% B (A: 0.1% formic acid in H_2_O, B: 0.1% formic acid in MeCN) over 9 min with 1 min gradient delay, 1 Hz acquisition rate at 254 nm. Data were processed with Compass DataAnalysis 5.1 (Bruker Daltonik). Purity is >98% of all final compounds.

The NO donor 3-cyano-5-nitropyridin-2-yl diethyldithiocarbamate (NOD) was synthesized from 0.5 g (2.73 mmol) of 2-chloro-5-nitronicotinonitrile and 0.7 g (3.13 mmol) of sodium diethyldithiocarbamate trihydrate in 12 ml of ethanol with refluxing for 1 h. The reaction mixture was cooled to room temperature and dissolved in 50 ml of water. The solid yellow precipitate was collected by filtration, washed with 30 ml of water, and re-crystallized from ethanol. This yielded 0.7 g (87%) of ND with the following characteristics: melting point 127–29 °С; mass (EI), *m/z* (*I*_relat_.(%)): 296.3707 [M]^+^ (61). C_11_H_12_N_4_O_2_S_2_. ^1^H NMR (DMSO-d_6_)*: d 1.13 (t, 3H, J = 7.2, CH_3_), 1.26 (t, 3H, J = 7.2, CH_3_), 3.83 (q, 2H, J = 7.1, NCH_2_), 4.32 (q, 2H, J = 7.1, NCH_2_), 8.76 (s, 1H, CH) and 9.79 (s, 1H, CH) ppm. ^13^C NMR (DMSO-d_6_): δ 187.31, 168.07, 152.43, 147.45, 137.05, 112.55, 108.72, 50.08, 48.46, 13.23 and 10.06 ppm.

The control compound 3-cyano-4,6-dimethyl-5-nitropyridin-2-yl piperidine-1-carbodithioate (CC) was synthesized by mixing 0.5 g (2.36 mmol) 3-cyano-4,6-dimethyl-5-nitropyridin-2-yl piperidine-1-carbodithioate and 0.6 g (2.74 mmol) sodium piperidine-1-carbodithioate dihydrate in 12 ml ethanol followed by reflux for 3 hours. Reaction mixture was cooled to room temperature and dissolved by 50 ml of water. A solid yellow precipitate was collected by filtration, washed in 30 ml water and re-crystallized from ethanol. The yield of CC was 0.64 g (80%). CC had the following characteristics: melting point 143–45 °С; mass (EI), *m/z* (*I*_relat_.(%)): 336.4346 [M]^+^ (47). C_14_H_16_N_4_O_2_S_2_. ^1^H NMR (DMSO-d_6_)*: d 1.62 (br m, 6H, (CH_2_)_3_), 2.64 (s, 3H, CH_3_), 2.74 (s, 3H, CH_3_), 4.02 (br m, 4H, N(CH_2_)_2_ ppm. ^13^C NMR (DMSO-d_6_): δ 190.48, 164.50, 161.92, 157.14, 150.24, 111.60, 109.71, 51.67, 26.17, 24.08, 22.85 and 19.28 ppm. * s—singlet, t— triplet, q—quartet.

#### X-ray diffraction study

Experimental intensities for compounds were collected on a Bruker SMART APEX3 (MoKα, *λ* = 0.71073 Å, graphite monochromator). The reflection intensities were corrected for absorption using the SADABS software. The structures were solved by a combination of direct methods and Fourier syntheses. Hydrogen atoms were calculated using geometrical restraints. All calculations were made using SHELXS and SHELXL. Crystal structures of compounds were deposited in the Cambridge Crystallographic Data Centre (CCDC no. 2301117 and 2301118).

### Treatment of Mtb and BCG with NO donors

For treatment experiments, Mtb or BCG was grown in 7H9 medium to OD_580nm_ of 0.8–1.0 at 37 °C with shaking at 100 rpm. Cells were diluted with fresh 7H9 medium to an OD_580nm_ of 0.1 in a total volume of 5 ml. NOD or CC were dissolved in DMSO and added to cultures to a final concentration of 100 µM which corresponds to 30× the minimum inhibitory concentration (MIC) of NOD. Cultures were incubated at 37 °C without shaking for up to 48 hours. Washing of NOD-treated mycobacteria did not improve growth on agar or liquid media and was not used routinely. In control experiments, Mtb was incubated with either 100 µM DETA/NO or 200 µM spermine NONOate or 10 mM sodium nitrite, pH 5.0 for up to 48 h.

### Detection of NO release in media and cells

NO release was estimated by the accumulation of its stable oxidation product, nitrite, using Griess reagent method^26^. NOD or CC or DETA/NO was incubated in 7H9 medium (pH 6.8) at a final concentration of 100 or 200 µM at 37 °C without shaking for 24 hours. Absorbance at 540 nm was measured at different time points. Known concentrations of sodium nitrite were used to construct a calibration curve. For determination of NO in mycobacterial lysates and spent media, BCG cultures were grown to OD_580_ ~ 0.8–1.0, centrifuged and resuspended in fresh medium (OD_580_ of 0.5). Independent cultures (10 ml) were set up in triplicate for each condition and treated with 200 µM NOD or CC or DMSO-only control (untreated control). At 1, 4, and 24 h, 2 ml aliquots were taken and centrifuged at 13,000×*g* for 5 min. The medium was removed and kept for nitrite determination; the pellets were washed with PBS and resuspended in 400 µl PBS containing Roche cOmplete™ Protease Inhibitor Cocktail and lysed by bead beating. Samples were centrifuged; supernatants and spent media were used for Griess Reagent System (Promega) by employing the internal standard provided by the kit. Intracellular concentrations of nitrite were normalized to protein content determined by Pierce™ BCA Protein Assay Kits.

For detection of NO in treated mycobacteria DAF-FM diacetate (4-Amino-5-Methylamino-2’,7’-Difluorofluorescein)^27^, purchased from Thermo Fisher Scientific, was used. BCG was pretreated with 10 µM of DAF-FM diacetate for 2 h, followed by washing with 0.85% (w/v) NaCl or 7H9 medium three times before treatment with NOD and CC (100 µM). For flow cytometry, BCG was incubated with either NOD or CC for 24 h, washed with 0.85% (w/v) NaCl, stained with 10 µM of DAF-FM for 1 h, followed by centrifugation and incubation in fresh 0.85% (w/v) NaCl for 15 min. DAF-FM diacetate-stained bacteria were analyzed in CytoFLEX flow cytometer and CytExpert software (Beckman Coulter), or fluorescence was measured in Varioskan Flash using filters for excitation 495 nm and emission 515 nm.

### Labeling of peptidoglycan

Mtb cultures were grown to OD_580nm_ 0.8–1.0 in the presence of tetracycline (20 ng/ml) and resuspended to OD_580nm_ 0.1 in 7H9 supplemented with tetracycline and fluorescent analog of D amino acid 7-hydroxycoumarincarbonylamino-D-alanine, HADA (1 mM) NOD or CC (100 µM) was added, before incubation of samples at 37 °C for 4 h without shaking, protected from the light. Following incubation, samples were washed 3 times with sterile PBS before fixing in 4% paraformaldehyde overnight, protected from the light. Aliquots were mounted onto slides for microscopic analysis. Slides were visualized using a Ti-Eclipse microscope (Nikon) equipped with a 12/10 bit high-speed Peltier-cooled CCD camera (FDI, Photonic Science), using the DAPI channel. Images were analyzed using NIS-Elements Imaging Software (Nikon).

### Transcriptomic analyses

RNA was extracted from three biological replicates of Mtb cultures after 4 h of treatment with either NOD or CC using the GTC/Trizol method^66^. After 4 h of NOD treatment, the bacteria retained culturability, thus enabling the study of adaptation during the transition to the DC state. RNA (2 µg) was labeled with Cy3 and Cy5 fluorophores and hybridized to an Mtb microarray as previously described^50^.

For quantitative RT-PCR, RNA was reverse transcribed to cDNA using SuperScript II reverse transcriptase kit (Thermo Fisher Scientific) with mycobacterial genome-directed primers^67^. The qPCRs were run in the Corbett Rotor*-*Gene 6000 (Qiagen) using the 2×SYBR green master mix (Thermo Fisher Scientific) and primers (Supplementary Table 1). Levels of expression were normalized to 16S rRNA. Relative gene expression in NOD-treated samples was calculated as the ratio of normalized gene copy number in NOD-treated samples to normalized gene copy number in CC-treated samples and expressed as relative Log_2_ fold change.

### Detection of Rpf peptides using mass-spectrometry

Mtb was grown in Sauton medium to OD_580_ 0.8. Filtered CSN was enriched on DEAE-Sepharose and digested with trypsin. LC–MS/MS was carried out using an RSLCnano HPLC system (Dionex, UK) and a Thermo Scientific LTQ Orbitrap Velos Mass Spectrometer. The raw data were processed using Proteome Discoverer (version 1.4.0.288, Thermo Scientific), Mascot (version 2.2.04, Matrix Science Ltd).

### Statistics and reproducibility

Unpaired *t*-test or one-way ANOVA (Prism 10) were used to evaluate the statistical differences in the growth and flow cytometry datasets. Differentially expressed genes in ND- compared to CC-treated cultures, 4 h after exposure, were identified using a modified *t*-test (GeneSpring 14.5; Agilent Technologies) with Benjamini and Hochberg multiple testing correction and defined as those with *p* < 1 × 10^−5^ and minimum fold change of 2.0. Numbers of samples and experiments are indicated in Figure legends.

### Reporting summary

Further information on research design is available in the Nature Portfolio Reporting Summary linked to this article.

## Supplementary information

Supplementary Information

Description of Additional Supplementary Materials

Dataset 1

Dataset 2

Dataset 3

Reporting Summary

## Data Availability

Microarray data have been deposited in the EBI BioStudies and are available under accession number E-MTAB-10776. Crystal structures of NOD and CC were deposited in the Cambridge Crystallographic Data Centre (CCDC no. 2301117 and 2301118). The source data underlying Figs. 1, 3, 4, 5, 6 can be found in Supplementary Dataset 3.
