# Supplementary material for: Nitric oxide induces the distinct invisibility phenotype of Mycobacterium tuberculosis
Source: Commun Biol. 2024 Sep 28;7:1206. doi: 10.1038/s42003-024-06912-0 (PMC11439070; doi:10.1038/s42003-024-06912-0)
Supplement: Supplementary file 1 — Supplementary Information [file 42003_2024_6912_MOESM1_ESM.pdf]

Nitric oxide induces the distinct invisibility phenotype of *Mycobacterium tuberculosis*

Brindha Gap-Gaupool[1]†, Sarah M. Glenn[1]†, Emily Milburn[1]†, Obolbek Turapov[1], Marialuisa Crosatti[1], Jennifer Hincks[2], Bradley Stewart[1], Joanna Bacon[3], Sharon L. Kendall[4], Martin I. Voskuil[5], Olga Riabova[6], Natalia Monakhova[6], Jeffrey Green[7], Simon J. Waddell[8*], Vadim A. Makarov[6*], Galina V. Mukamolova[1,9*]

[1]Leicester Tuberculosis Research Group, Department of Respiratory Sciences, University of Leicester, Leicester, LE1 9HN, UK; [2]FACS Facility Core Biotechnology Services, University of Leicester, Leicester, LE1 9HN, UK; [3]Discovery Group, Vaccine Development and Evaluation Centre, UK Health Security Agency, Porton Down, SP4 0JG, UK; [4]Centre for Endemic, Emerging and Exotic Disease, the Royal Veterinary College, Hatfield, Hertfordshire, AL9 7TA, UK; [5]Department of Immunology and Microbiology, University of Colorado Anschutz Medical Campus, Aurora, Colorado, USA; [6]Research Center of Biotechnology, Russian Academy of Sciences, Moscow, Russia; [7]School of Biosciences, University of Sheffield, Sheffield, S10 2TN, UK; [8]Global Health and Infection, Brighton and Sussex Medical School, University of Sussex, Brighton, BN1 9PX, UK; [9] The National Institute for Health and Care Research Leicester Biomedical Research Centre, University of Leicester, Leicester, LE1 9HN, UK.

†These authors contributed equally.

*Corresponding authors:
Simon J. Waddell: S.Waddell@bsms.ac.uk
Vadim A. Makarov: makarov@inbi.ras.ru
Galina V. Mukamolova: gvm4@leicester.ac.uk

This file contains
Supplementary Figures 1-8
Supplementary Table 1

Supplementary figures

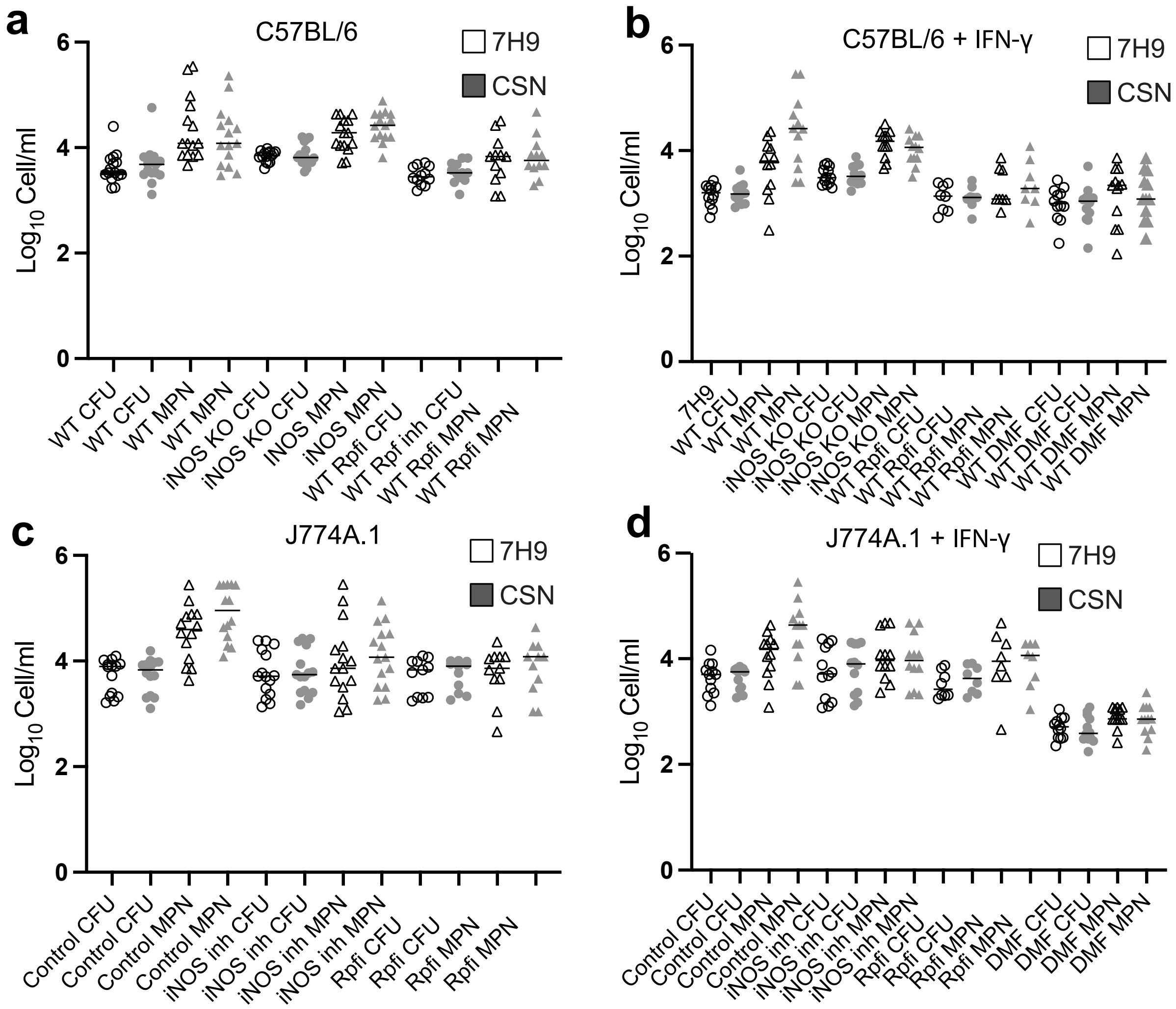

Supplementary Figure 1. CFU and MPN counts of Mtb recovered from infected murine macrophages. Murine macrophages were infected with Mtb at MOI of 1 for 24 hours prior to CFU and MPN counting in 7H9 or CSN. The Rpf inhibitor (Rpfi) was added to resuscitation media. **a**, **b** C57BL/6 wild type (WT) and iNOS knockout (iNOS KO. **a** untreated C57BL/6, **b** IFN-γ treated C57BL/6. **c**, **d** J774A.1 cells, untreated control (**c**) or IFN-γ treated cells (**d**). iNOS was inhibited by addition of aminoguanidine (iNOS inh). Data are means ± SEM for at least twelve biological replicates from four experiments. Chemical concentrations used: IFN-γ, 10 ng/ml; aminoguanidine, 500 μM; DMF, 25 μM; Rpfi, 35 μM.

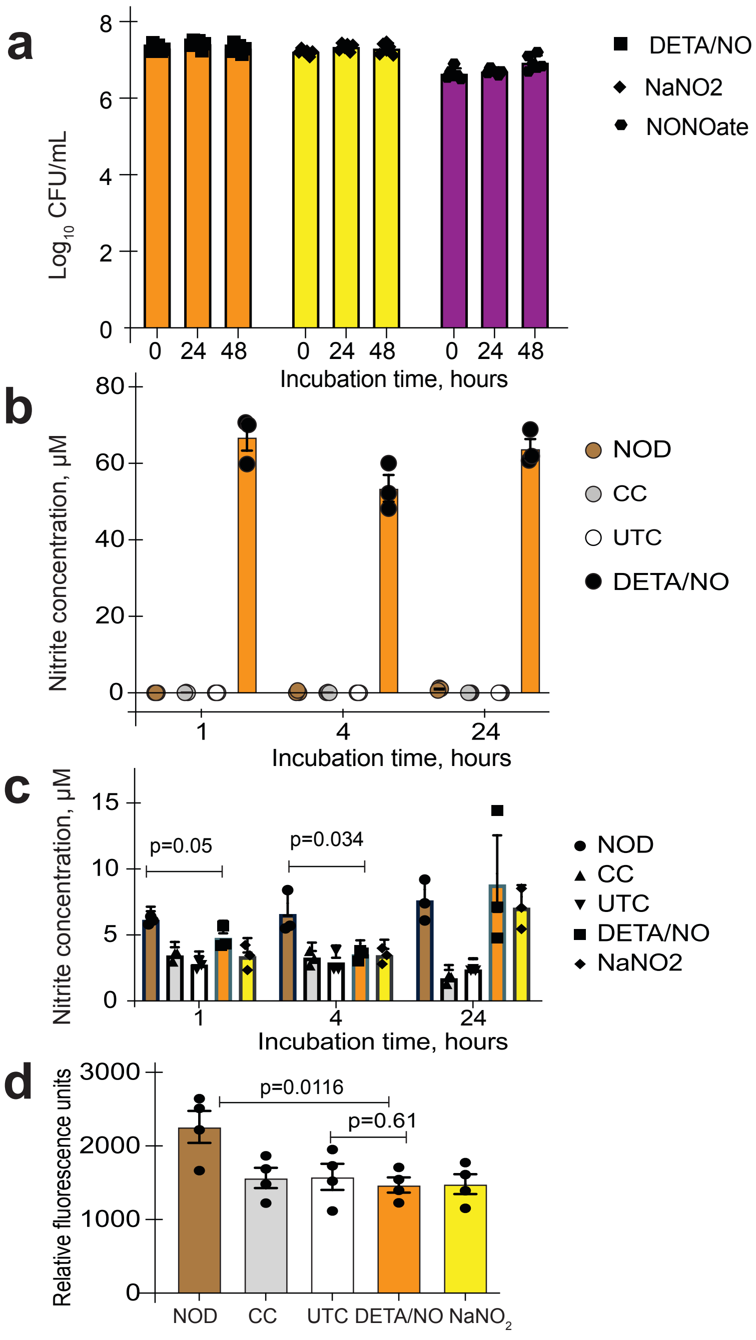

Supplementary Figure 2. Commercially available NO donors or acidified nitrite did not impact of CFU counts of mycobacteria. a Mtb was incubated with either 100 µM DETA NONOate (DETA/ NO) or 200 µM spermine NONOate (NONOate) or 10 mM sodium nitrite ($NaNO_2$), pH 5.0 for up to 48 hours. CFU counts were measured at 0, 24 and 48 hours. Data are means ± SEM (n=6 from 2 independent experiments). **b**, **c** Nitrite concentrations in media were assessed using Griess reagent by measuring absorbance at 540 nm in spent media (**b**) or BCG lysates (**c**). NOD, DETA/NO, and CC were added to 7H9 medium at final concentrations of 100 µM and incubated for 24 hours. Data are means ± SEM for 6 biological replicates obtained in two experiments. **d** Detection of NO in BCG using DAF-FM diacetate and fluorescence measurement. Bacteria were either untreated control (UTC) or incubated with NOD, CC, $NaNO_2$ or DETA/NO for 1 hour. Data are means ± SEM for 4 biological replicates from one representative experiment; p-values are for unpaired t-test.

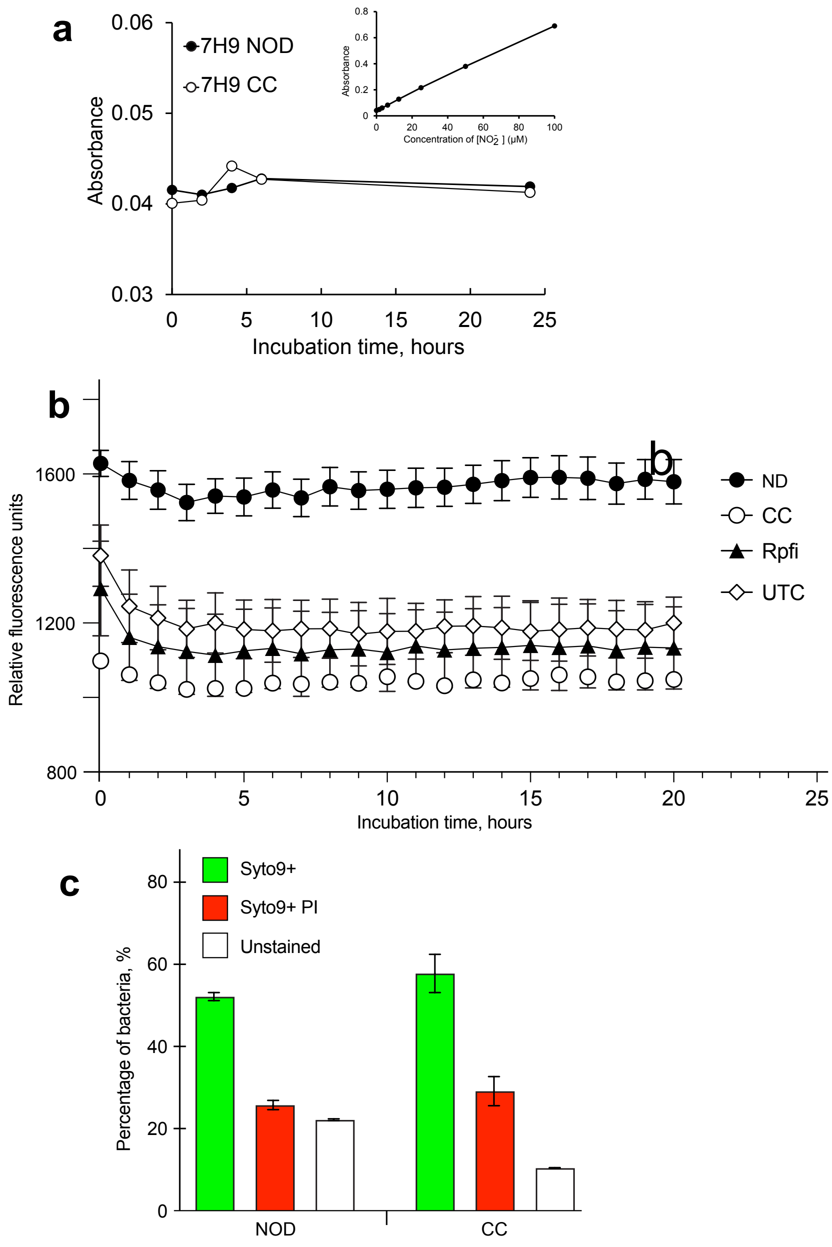

Supplementary Figure 3. NOD treatment resulted in release of NO in BCG as judged by increased fluorescence of DAF-FM diacetate and did not impact on BCG viability assessed by LIVE/DEAD staining. a Griess reagent was applied to assess nitrite concentration by measuring absorbance at 540 nm. Known concentrations of $NaNO_2$ were used for making a calibration curve (top right corner). **b** DAF-FM diacetate preloaded bacteria were incubated with either NOD, CC, or Rpf inhibitor (Rpfi) for 20 hours; fluorescence was measured every hour in Varioskan Flash plate reader at excitation/emission 495/515 nm. **c** BCG was treated with NOD or CC for 24 hours followed by dual staining with PI and SYTO 9 and analysis by flow cytometry. SYTO 9 - excitation/emission at 480/500 nm; PI excitation/emission at 490/635 nm. The same chemical concentrations were used in all experiments: NOD or CC –100 µM, – Rpfi – 35 µM, DAF-FM diacetate – 10 µM. Data are means ± SEM for four (**a**, **b**) or three (**c**) biological replicates from one representative experiment.

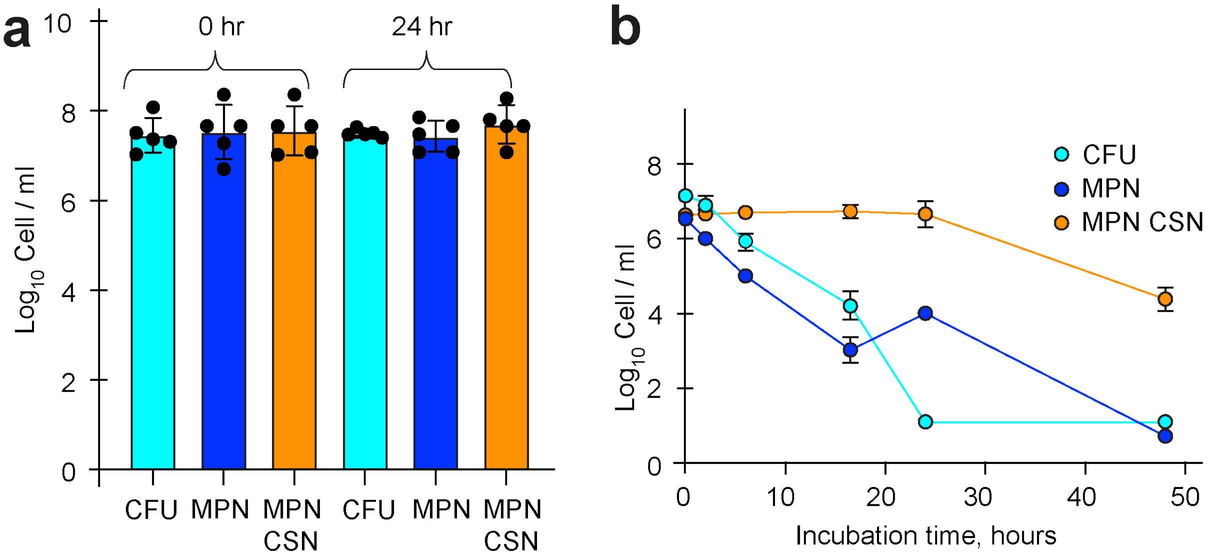

Supplementary Figure 4. CC and NOD had different effects on Mtb CFU, MPN 7H9 and MPN CSN counts. Mtb were treated with 100 μM CC (**a**) or NOD (**b**). Data are means ± SEM for six (**a**) and three (**b**) biological replicates from two and one experiments, respectively.

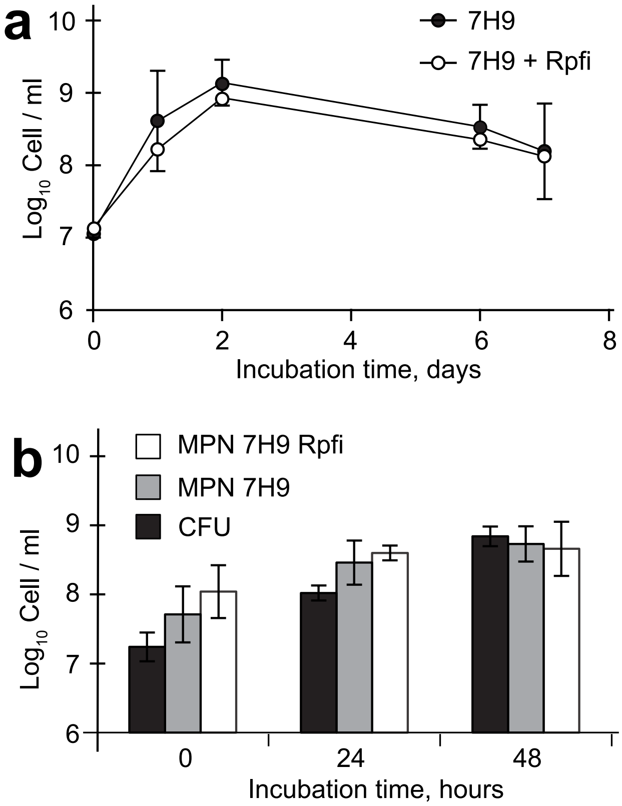

Supplementary Figure 5. The Rpf-inhibitor (Rpfi) did not impact on Mtb growth. a Mtb was grown in 7H9 or 7H9+Rpfi (35 μM) in flasks with shaking (100 rpm). CFU counts were determined at 0, 1, 2, 6, 7 days. **b** Mtb was grown in microplates used for MPN assays. Pre-diluted actively growing Mtb cultures were incubated in 7H9 or 7H9+Rpfi (35 μM); CFU, MPN_7H9 and MPN_7H9+Rpfi counts were assessed after 0, 24 and 48 hours of incubation. **a, b** Data are means ±95% confidence intervals for two biological replicates from one experiment.

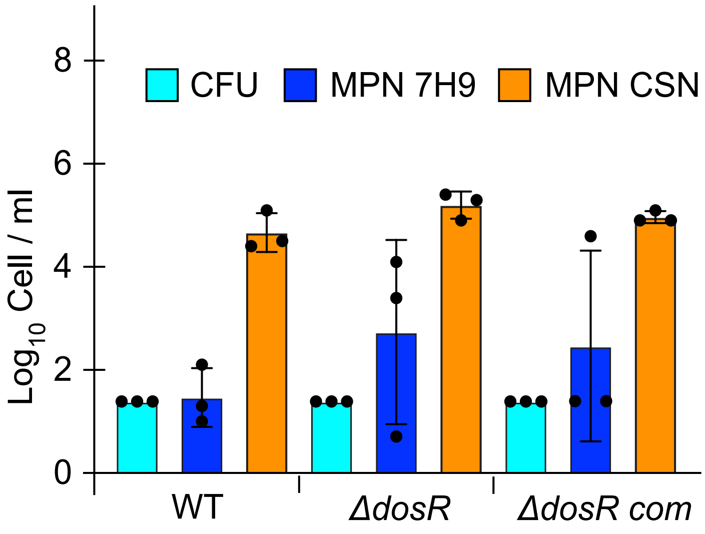

Supplementary Figure 6. The wild type (WT), *dosR* deletion mutant (Δ*dosR*), and complemented *dosR* deletion mutant (Δ*dosR com*) deletion of Mtb were equally resuscitated in CSN-supplemented medium. There was no statistically significant difference between CFU, MPN and MPN_CSN counts obtained for the wild type (WT), Δ*dosR* and Δ*dosR com* cultures treated with NOD for 24 hours ($p>0.05$, one-way ANOVA). Data are means ±SEM for three biological replicates from one experiment.

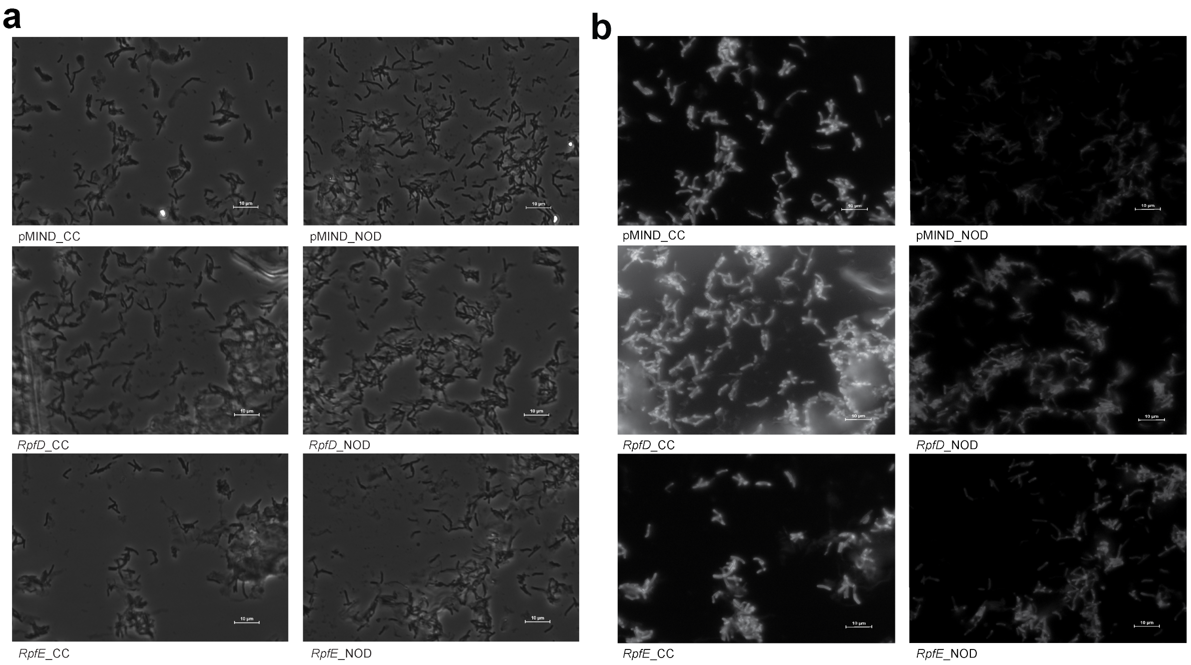

Supplementary Figure 7. Representative microscopy images used for analysis of HADA labelled Mtb. Mtb were incubated with 1 mM HADA and either CC (left panels) or NOD (right panel) for 4 hours. **a** Phase contrast microscopy; **b** fluorescence microscopy (DAPI channel).

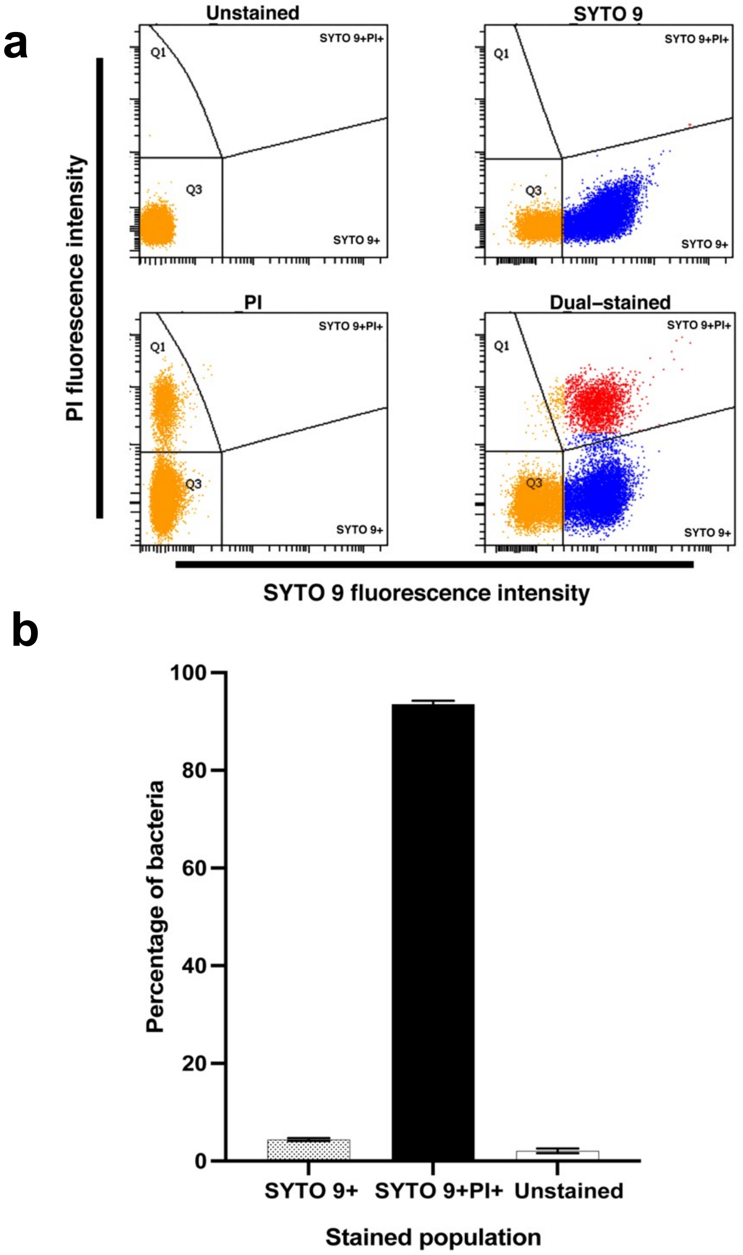

Supplementary Figure 8. Detection of *M. bovis* BCG populations using live/dead staining. **a** Gating strategy used in this study. Four different *M. bovis* BCG samples: unstained, single-stained with either SYTO 9 or propidium iodide (PI) and dual-stained with both SYTO 9 and PI were generated after ND-treatment. Each sample was run using a FACs-Aria flow cytometer using the following parameters: SYTO 9 excitation/emission at 480/500nm and PI excitation/emission at 490/635nm. **b** The percentage of unstained, SYTO 9+ and SYTO 9+PI+ bacteria were quantified in a sample of heat-killed *M. bovis* BCG as a control.

Supplementary Datasets

Dataset 1. Differentially expressed genes in NOD-treated Mtb vs CC-treated Mtb. Mtb were treated with NOD or CC for 4 hours prior isolation of RNA. (Microsoft Excel file)

Dataset 2. Identification of regulatory pathways in NOD-treated Mtb using the Transcription Factor Overexpression tool. (Microsoft Excel file)

Dataset 3. Numerical source data. (Microsoft Excel file)

Supplementary Table 1. Primers used for RT-qPCR assessment of gene expression.

Primer name	Primer sequence (5´-3´)
16S rRNAF	GAGATACTCGAGTGGCGAAC
16S rRNAR	GGCCGGCTACCCGTCGTC
rpfBF	TCGGATCAAGAAGGTCACCG
rpfBR	GCTACCGCGAACGTCACATC
rpfCF	AGCTGCCTCTCGGAACAAC
rpfCR	GACCACAGTGCGATCGGAAG
rpfDF	GCAACAGATCGAGGTCGCAG
rpfDR	CGAGGAACGTCAGGATGTGG
rpfEF	TGGCCTACAGCGTGAACTGG
rpfER	GAACGCAGCACGTTCTAGC
lsr2F	TACTTCCAATCCATGGCGAAG
lsr2R	TATCCACCTTTACTGTCAGGTC
cwlMF	ATATCGGCTACATCACCAACC
cwlMR	GTTCTTGCCTAACAGATACAGCC
bfrBF	TAACGAATTCACAGCGGCAC
bfrBR	CACGAGCATCATTGCATGGTT
mbtLF	ATGTGGCGATATCCACTAAGTACA
mbtLR	GTCAGGTCAATGTTGAGGTCGT
ideRF	ACGAGTAACCGTCGAAACCA
ideRR	TCAGACTTTCTCGACCTTGACC
thiXF	ATCGAAGATCAACGTGGTGGG
thiXR	ACCGTCGTGCATTTCAGGG
frdAF	TGGCTGTGTGACCAAGATGC
frdAR	GAAACAACGTGTGCAGGAGG
frdBF	GCGGCAGTAGTGGTATGACG
frdBR	GCCATGAAGTCACTGATGTCG
frdCF	TGCTGTTACCTGGTTCGGATCG
frdCR	ACCATCCAGGCAACGATCACC
murIF	CTGTACGCACTATCCACTGC
murIR	GACGCAATAAGTCGATCTCGG
sigHF	GCAACGCTTCTAACGCTTCG
sigHR	ACCGGATACTGACCAACACC
