# Supplementary material for: Nitric oxide induces the distinct invisibility phenotype of Mycobacterium tuberculosis
Source: Commun Biol. 2024 Sep 28;7:1206. doi: 10.1038/s42003-024-06912-0 (PMC11439070; doi:10.1038/s42003-024-06912-0)
Supplement: Supplementary file 2 — Description of Additional Supplementary Materials [file 42003_2024_6912_MOESM2_ESM.pdf]

Description of Additional Supplementary Files

File name: Dataset 1

Description: Differentially expressed genes in NOD-treated Mtb vs CC-treated Mtb. Mtb were treated with NOD or CC for 4 hours prior isolation of RNA.

File name: Dataset 2

Description: Identification of regulatory pathways in NOD-treated Mtb using the Transcription Factor Overexpression tool.

File name: Dataset 3

Description: Numerical source data.
